# Laparoscopic vs. open portoenterostomy for biliary atresia: a meta-analysis of pediatric surgical outcomes

**DOI:** 10.3389/fped.2024.1476195

**Published:** 2024-12-09

**Authors:** Jie Zhu, Bin Wu, Peng Cai, Jiang Pan, Zhenwei Zhu

**Affiliations:** Department of General Surgery, Children’s Hospital of Soochow University, Suzhou, China

**Keywords:** biliary atresia, portoenterostomy, laparoscopy, children, surgery, treatment

## Abstract

**Background:**

The pivotal importance of surgical treatment for pediatric biliary atresia is well-established. This systematic review and meta-analysis was designed to assess the comparative efficacy and safety of open portoenterostomy (OPE) and laparoscopic portoenterostomy (LPE) in managing this condition, providing valuable guidance for clinical decision-making.

**Methods:**

A comprehensive literature review was conducted by two researchers in databases such as PubMed, up to July 10, 2024, focusing on studies that evaluated the role of LPE vs. OPE. Data analysis was performed utilizing the RevMan 5.4 software suite.

**Results:**

The meta-analysis incorporated findings from 15 studies involving a total of 964 pediatric patients with biliary atresia. LPE was associated with decreased intraoperative blood loss [Mean Difference (MD) = −10.80, 95% Confidence Interval (CI) (−13.54, −8.05)] and shortened hospital stay [MD = −2.18, 95% CI (−3.69, −0.67)]. Conversely, the operative time for LPE was considerably longer when compared to OPE [MD = 35.45, 95% CI (26.17, 44.72)]. No significant disparities were noted in the postoperative jaundice clearance rate [Odds Ratio (OR) = 0.98, 95% CI (0.71, 1.35)], incidence of postoperative cholangitis [OR = 0.96, 95% CI (0.66, 1.39)], the rate of liver transplantation between the two surgical approaches [OR = 0.69, 95% CI (0.32, 1.48)], or 2-year survival of the native liver [OR = 1.10, 95% CI (0.67, 1.80)].

**Conclusion:**

LPE offers more advantages over OPE, including diminished invasiveness and expedited recovery. These benefits suggest that LPE is an emerging and viable alternative in the clinical management of biliary atresia, warranting further investigation and consideration in surgical practice.

## Introduction

Biliary atresia is characterized by intrahepatic cholestasis resulting from the sclerosis and obstruction of the bile ducts (1). This condition can arise from a multitude of etiologies, including viral infections and the progressive exacerbation of hepatic fibrosis (2). The pathogenesis of biliary atresia encompasses a complex interplay of factors that lead to the obliteration of the bile ducts, thereby impeding the normal flow of bile and causing the characteristic symptoms of the disease (3). Currently, biliary atresia stands as the predominant cause of obstructive jaundice in neonates, representing a significant clinical challenge in pediatric hepatology (4). In the absence of effective treatment, biliary atresia can inexorably progress to liver failure and ultimately result in mortality (5). At present, the incidence of biliary atresia is about 0.5–1.510000 which is higher in Asia (6). The cornerstone of biliary atresia management is surgical intervention. The timing of surgery is intricately linked to patient outcomes, with earlier intervention correlating to less severe hepatic fibrosis (7). Previous study (8) has shown that performing the surgery at a younger age, specifically within the first 60 days postpartum, is associated with improved bile drainage efficacy. Consequently, the initiation of early surgical treatment for biliary atresia exerts a significant influence on the clinical outcomes for pediatric patients.

In 1959, Dr. Morio Kasai pioneered the use of the Kasai procedure, known formally as portoenterostomy, to successfully treat patients with intrahepatic biliary atresia. This groundbreaking surgical technique marked a significant advancement in the field, substantially enhancing the survival and cure rates for individuals afflicted with this condition (9). The Kasai portoenterostomy demands a high level of technical skill and precision, given its complexity and the critical nature of the procedure in restoring bile flow and mitigating the progression of liver disease (10, 11). The requirement is to meticulously analyze the hilar lesions to ensure the atresia bile duct is opened to its fullest extent without causing any damage (12). Additionally, it is imperative to undertake the reconstruction of both the bile duct and the intestinal tract (13). Kasai procedure is a proven therapeutic approach for biliary atresia, encompassing both the conventional open portoenterostomy (OPE) and the laparoscopic portoenterostomy (LPE). With the swift advancement of minimally invasive surgical techniques, laparoscopic surgery has seen significant growth in recent years (14). As a form of minimally invasive intervention, laparoscopic surgery has expanded its scope of application beyond that of traditional open procedures. It offers several benefits, including minimal scarring, accelerated postoperative recovery, and reduced trauma (15, 16). Nevertheless, the comparative effectiveness of LPE and OPE in managing biliary atresia remains a subject of debate and lacks clarity. Consequently, this meta-analysis aims to assess the efficacy and safety of both LPE and OPE as treatments for biliary atresia, thereby offering valuable insights to guide clinical practice in this area.

## Methods

This meta-analysis was performed according to the Preferred Reporting Items for Systematic reviews and Meta-Analyses (PRISMA) statement (17).

### Literature search

The search was conducted from the inception of the databases up to July 10, 2024. The databases covered in this meta-analysis were Pubmed, Cochrane Library, Web of Science, Embase, Clinical Trials, China National Knowledge Infrastructure (CNKI), Wanfang, and VIP databases. Our approach to literature retrieval involved a combination of subject-specific and free terms, conducted across both Chinese and English databases. Additionally, we employed the “snowball” technique to explore references and related reviews cited within the identified literature. The search was restricted to literature available in English and Chinese languages.

### Inclusion and exclusion criteria

The criteria for inclusion in this meta-analysis were as follows: The study subjects had been diagnosed with biliary atresia and had undergone surgical treatment, regardless of their nationality, gender, or ethnicity. The study types eligible for inclusion were cohort studies and case-control studies. The studies had to compare the efficacy and safety of LPE and OPE. The studies should have reported relevant outcome measures, such as intraoperative blood loss, hospital stay duration, surgical duration, postoperative jaundice clearance rate, postoperative cholangitis incidence, liver transplantation rate, and survival with the native liver.

The exclusion criteria for this meta-analysis were as follows: Studies that were classified as conference abstracts, case reports, and systematic reviews were excluded from the analysis. Studies for which full-text articles or requisite outcome data were inaccessible, despite attempts to contact the authors for additional information, were not included. Articles identified as duplicates or repetitive publications within the literature search were excluded to maintain the integrity of the analysis.

### Literature screening and data extraction

In accordance with the established inclusion criteria, two researchers independently conducted a preliminary screening of the literature by reviewing titles and abstracts. Subsequently, a detailed data extraction was performed through a full-text review to identify and exclude studies that did not fulfill the criteria. The final selection of included literature was verified by cross-checking between the two researchers. Any discrepancies were resolved through discussion to reach a consensus.

The data extracted from the eligible literature encompassed key information such as the author(s), year of publication, sample size, patient age, body weight, disease progression, intervention methods, and the specific outcome measures assessed.

### Literature quality evaluation

The quality assessment of the included studies was conducted using the Newcastle-Ottawa Scale (NOS) (18) a widely recognized tool for evaluating the methodological quality of non-randomized studies in meta-analyses. The NOS criteria encompass three domains: selection of participants, comparability of study groups, and assessment of outcomes. The scale assigns a maximum of nine stars, with higher scores indicating better quality. Discrepancies in the quality assessment were resolved through a collaborative deliberation process, culminating in a consensus on the final quality rating for each study. This approach ensured a standardized and transparent evaluation of study quality within the meta-analysis.

### Statistical analysis

For the execution of this meta-analysis, we utilized the RevMan 5.4 software. Initially, we conducted an assessment of heterogeneity among the included studies. A study was considered homogeneous if the *P*-value was ≥ 0.1 and the *I*^2^ statistic was ≤ 50%. Under these criteria, a fixed-effect model was utilized for the meta-analysis. In contrast, the presence of heterogeneity was indicated by a *P*-value < 0.1 or an *I*^2^ statistic > 50%, prompting the application of a random-effects model. The continuous outcome measures were reported as the mean difference (MD), while the dichotomous outcome measures were expressed as the odds ratio (OR). For all analyses, we calculated the 95% confidence intervals (CIs) to quantify the precision of the estimated effects. To evaluate the susceptibility to publication bias, we performed a funnel plot analysis and Egger's regression test. Additionally, we undertook sensitivity analyses through the sequential exclusion of each study, thereby gauging the individual contribution of each to the aggregated outcomes. A statistically significant result was considered when the *P*-value was less than 0.05, indicating a difference between the groups with respect to the presence of publication bias.

## Results

**Figure 1:**
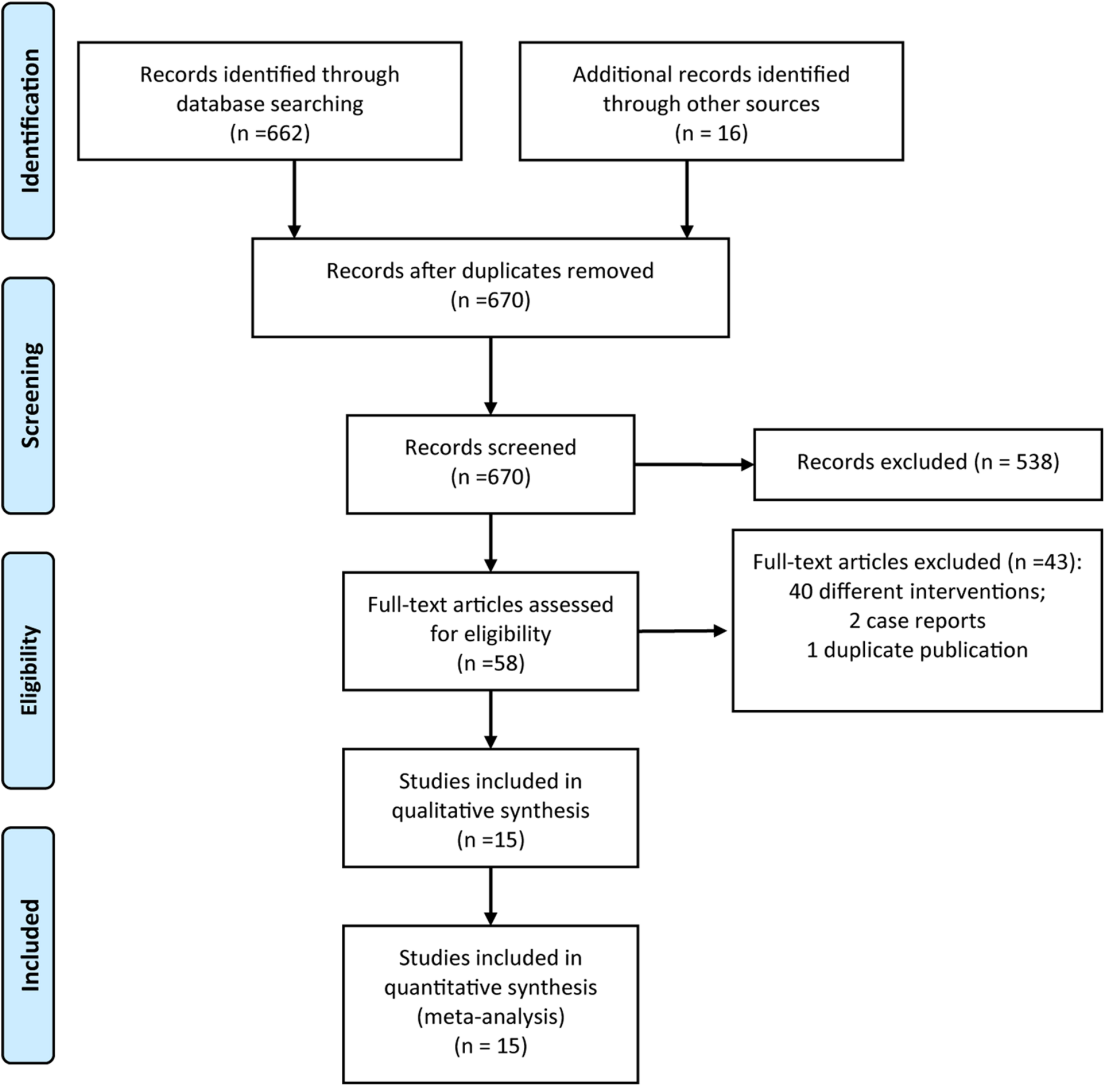
Flow diagram of study inclusion.

From the initial database search, a total of 678 articles were identified. As depicted in Figure 1, following a systematic process that included Endnote de-duplication, title and abstract screening, and full-text review, a total of 15 studies (19–33) were ultimately deemed eligible for inclusion in accordance with the established criteria.

A total of 964 children with biliary atresia were included in 15 studies (19–33), including LPE group (*n* = 439) and OPE group (*n* = 525). 5 reports documented modification of the classic Kassai. The refinements included more delicate dissection of the fibrous tissue at the porta hepatis, as well as improvements in the anastomotic techniques between the jejunum and the hilum. These technical adjustments aim to reduce trauma during the surgical procedure, facilitate smooth bile flow, and thereby enhance the rate of jaundice resolution. Studies (28, 33) have introduced novel antireflux mechanisms, such as the utilization of valved intestinal segments, to diminish the occurrence of postoperative cholangitis. Only three studies (23, 27, 28) reported the incidence of failed surgical procedures and the need for redo surgery. The basic information included in the literature is shown in Table 1.

**Table 1:** The characteristics of included RCTs.

Study ID	Sample size		Gender (male/female)		Age (days)		Weight (kg)		Follow-up period (months)	
	LPE group	OPE group	LPE group	OPE group	LPE group	OPE group	LPE group	OPE group	LPE group	OPE group
Aspelund et al. (19)	5	24	NR	NR	68.6 ± 31.5	70.0 ± 16.1	NR	NR	9.9	40
Chan et al. (20)	16	16	5/11	6/10	65.6 (45–106)	48.9 (34–73)	NR	NR	60	60
Chi and Chen (21)	43	42	NR	NR	NR	NR	NR	NR	12	12
Hou et al. (22)	26	42	13/13	19/23	78.4 ± 9.1	86.8 ± 11.9	5.0 ± 0.4	5.3 ± 0.5	26.9 ± 10.3	28.4 ± 8.3
Liu (23)	42	42	19/23	22/20	74.32 ± 20.20	81.57 ± 14.93	5.2 ± 0.5	5.3 ± 1.0	3	3
Murase et al. (24)	12	65	3/9	26/39	53 (41–77)	65.5 (29–119)	4.6 (2.464–6.755)	4.6 (2.164–6.755)	NR	NR
Nakamura et al. (25)	17	14	NR	NR	65.5 (29–119)	69.3 (29–100)	4.4 (3.2–6.2)	4.1 (2.2–6.2)	25.2–37.2	25.2–37.2
Oetzmann et al. (26)	8	11	6/2	5/6	NR	NR	NR	NR	NR	NR
Sun et al. (27)	48	47	25/23	24/23	68.1 ± 19.55	67.09 ± 18.14	NR	NR	12	12
Sun et al. (28)	44	47	21/23	24/23	64.5 ± 20.41	68.34 ± 17.59	NR	NR	16	17
Ure et al. (29)	12	28	6/6	14/14	57 ± 27	57 ± 21	NR	NR	24	24
Wada et al. (30)	12	11	NR	NR	65.8 (29–119)	64.7 (29–100)	4.2 (3.2–5.0)	4 (2.2–5.7)	35.3 (3–60)	45.1 (1–91)
Zhang et al. (31)	55	48	26/29	23/25	64. 50 ± 20. 41	68. 34 ± 17.59	NR	NR	30	30
Zhou et al. (32)	43	43	30/13	31/12	72.5 ± 7.9	71.3 ± 8.1	NR	NR	24	24
Zhu et al. (33)	56	45	30/26	24/21	62.1 ± 8.4	58.5 ± 8.2	4.8 ± 1.5	4.6 ± 1.4	18.5 ± 2. 3	24.3 ± 2.6

LPE, laparoscopic portoenterostomy; OPE, open portoenterostomy; NR, not reported.

For the quality assessment, the quality score of the included studies, assessed by the NOS scale, ranged from 7 to 8 (Table 2).

**Table 2:** The NOS score of included studies.

Study	Patient selection	Comparability	Outcome assessment	NOS total score
Aspelund et al. (19)	3	2	2	7
Chan et al. (20)	3	2	3	8
Chi and Chen (21)	3	2	2	7
Hou et al. (22)	3	2	3	8
Liu (23)	3	2	2	7
Murase et al. (24)	3	2	2	7
Nakamura et al. (25)	3	2	3	8
Oetzmann et al. (26)	3	2	2	7
Sun et al. (27)	3	2	2	7
Sun et al. (28)	3	1	3	7
Ure et al. (29)	3	2	2	7
Wada et al. (30)	3	2	2	7
Zhang et al. (31)	3	2	2	7
Zhou et al. (32)	3	2	2	7
Zhu et al. (33)	3	2	3	8

### Meta-analysis

**Figure 2:**
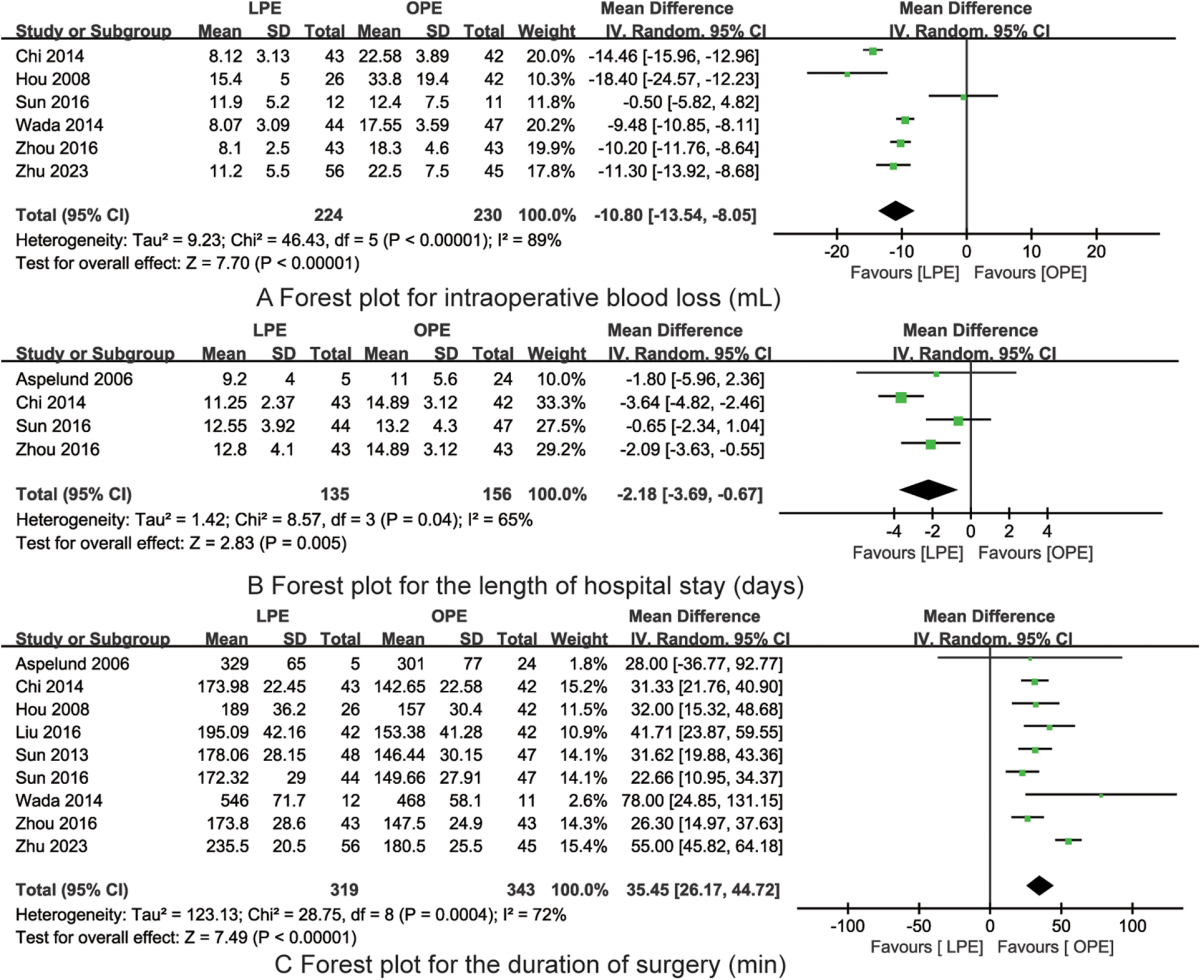
The forest plots for intraoperative blood loss **(A)**, length of hospital stay **(B)** and duration of surgery **(C)** (LPE is associated with reduced intraoperative blood loss, decreased length of hospital stay but longer duration of surgery).

Six studies provided data on the impact of LPE vs. OPE on intraoperative blood loss in pediatric patients with biliary atresia. An assessment of heterogeneity revealed a high degree of variability among these studies (*I*^2^ = 89%, *P* < 0.001). Consequently, a random-effects model was selected for the meta-analysis. The meta-analysis results indicated that the intraoperative blood loss in the LPE group was significantly reduced compared to the OPE group, with a statistically significant difference [MD = −10.80, 95% CI (−13.54, −8.05), *P* < 0.001, Figure 2A].

In the evaluation of the impact of LPE vs. OPE on the length of hospital stay for children with biliary atresia, data from four studies were considered. Heterogeneity was observed among these studies, as indicated by an *I*^2^ value of 65% and a *P*-value of 0.04. Given the presence of heterogeneity, a random-effects model was employed for the meta-analysis. The findings from the meta-analysis demonstrated that the length of hospital stay for the LPE group was significantly shorter than that for the OPE group, with the difference being statistically significant [MD = −2.18, 95% CI (−3.69, −0.67), *P* = 0.005, Figure 2B].

Nine studies contributed data on the comparative duration of surgery for LPE vs. OPE in children with biliary atresia. An assessment of heterogeneity among these studies revealed a substantial level of inconsistency (*I*^2^ = 72%, *P* < 0.001). In light of this heterogeneity, a random-effects model was selected for the meta-analysis. The meta-analysis indicated that the duration of surgery in the LPE group was significantly longer than in the OPE group, with the observed difference being statistically significant [MD = 35.45, 95% CI (26.17, 44.72), *P* = 0.005, Figure 2C].

**Figure 3:**
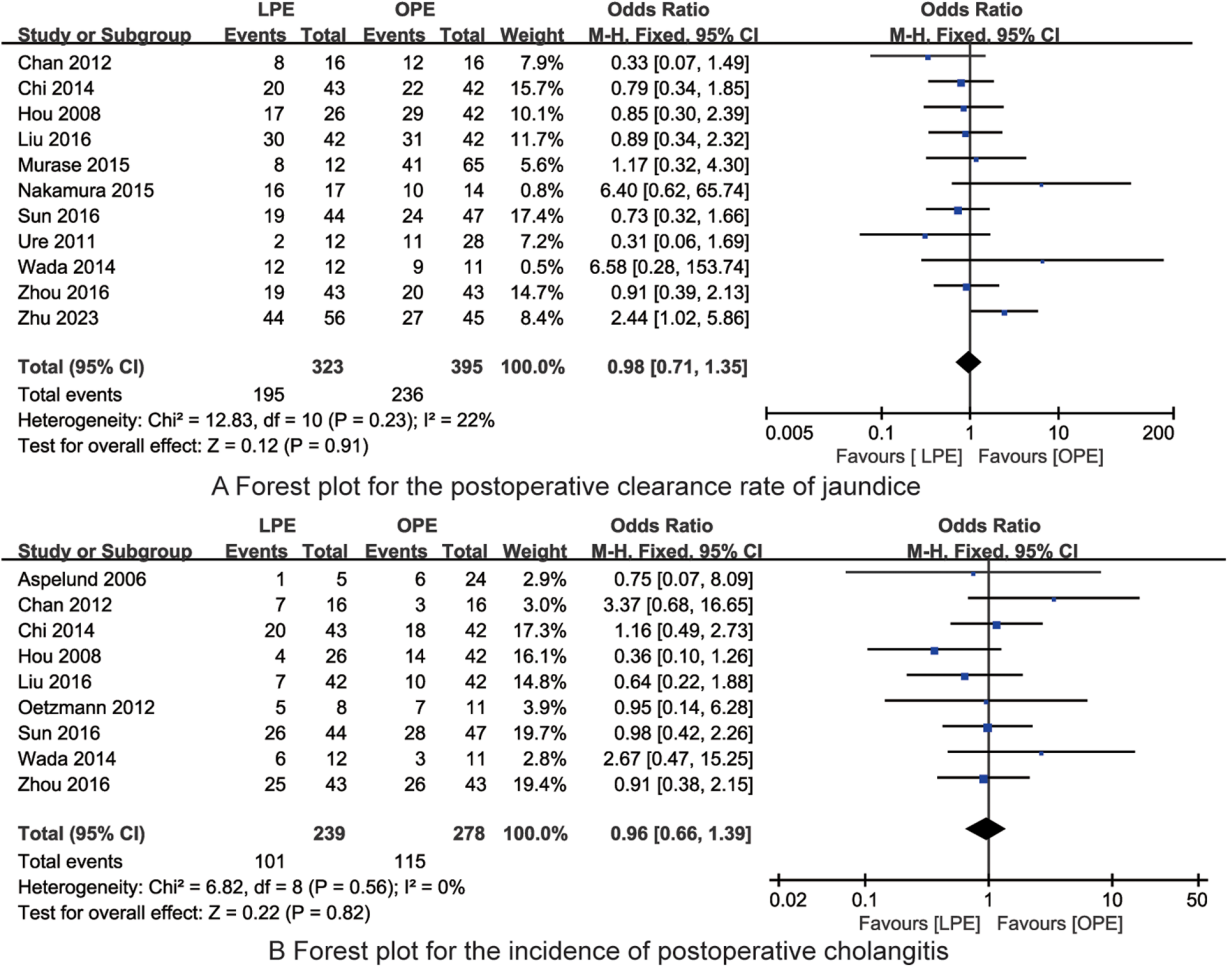
The forest plots for the postoperative clearance rate of jaundice **(A)** and incidence of postoperative cholangitis **(B)** (no significant differences in the postoperative clearance rate of jaundice and the incidence of postoperative cholangitis between LPE and OPE are found).

Eleven studies were identified that assessed the impact of LPE vs. OPE on the postoperative clearance rate of jaundice in children with biliary atresia. Upon evaluation, the studies exhibited no significant heterogeneity (*I*^2^ = 22%, *P* = 0.23). Consequently, a fixed-effects model was deemed appropriate for the meta-analysis. The meta-analysis results did not reveal a statistically significant difference in the postoperative clearance rate of jaundice between the LPE and OPE groups [OR = 0.98, 95% CI (0.71, 1.35), *P* = 0.91, Figure 3A].

In the analysis of the impact of LPE vs. OPE on the incidence of postoperative cholangitis in children with biliary atresia, data from nine studies were included. The studies displayed no significant heterogeneity (*I*^2^ = 0%, *P* = 0.56). As a result, a fixed-effects model was utilized for the meta-analysis. The findings from the meta-analysis indicated no statistically significant difference in the incidence of postoperative cholangitis between the LPE and OPE groups [OR = 0.96, 95% CI (0.66, 1.39), *P* = 0.82, Figure 3B].

**Figure 4:**
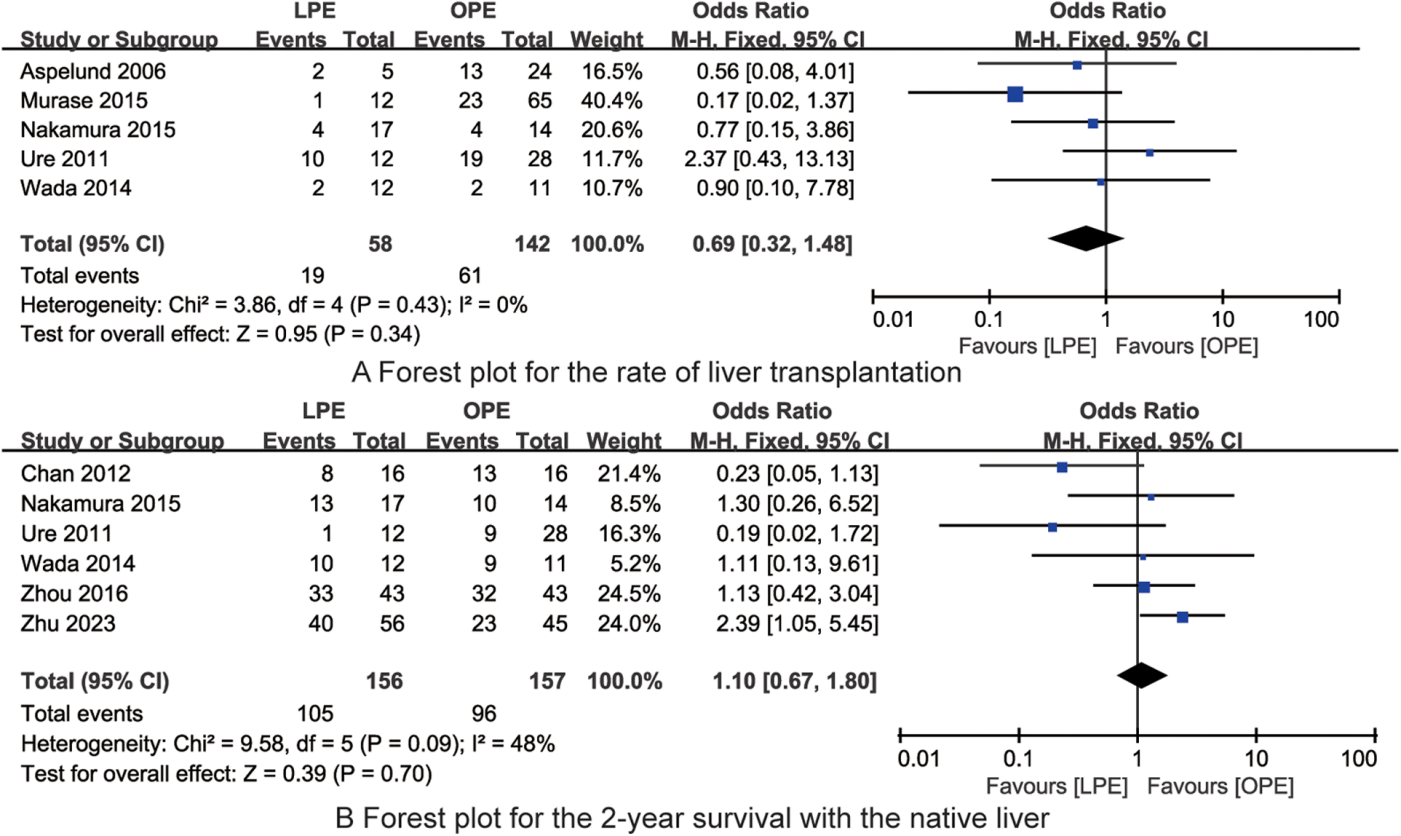
The forest plots for the rate of liver transplantation **(A)** and 2-year survival with the native liver **(B)** (no significant differences in the rate of liver transplantation and 2-year survival with the native liver between LPE and OPE are found).

In the examination of the impact of LPE vs. OPE on the rate of liver transplantation in children afflicted with biliary atresia, findings from five studies were incorporated. An assessment for heterogeneity among these studies yielded no significant variation (*I*^2^ = 0%, *P* = 0.43). Accordingly, a fixed-effects model was selected for the meta-analysis. The meta-analytic findings did not demonstrate a statistically significant difference in the rate of liver transplantation between the LPE and OPE groups [OR = 0.69, 95% CI (0.32, 1.48), *P* = 0.34, Figure 4A].

In the evaluation of the impact of LPE vs. OPE on the 2-year survival rate with the native liver in children with biliary atresia, data from six studies were analyzed. The heterogeneity assessment among these studies indicated a moderate level of variability (*I*^2^ = 48%, *P* = 0.09). The meta-analysis revealed no statistically significant difference in the 2-year survival rate with the native liver between the LPE and OPE groups [OR = 1.10, 95% CI (0.67, 1.80), *P* = 0.70, Figure 4B]. This suggests that both surgical approaches are comparable in terms of 2-year survival outcomes for children with biliary atresia.

### Sensitivity analysis

Upon thorough review, it was found that the exclusion of any individual study did not significantly affect the overall findings, thereby indicating the robustness of the meta-analytic conclusions.

### Publication bias

**Figure 5:**
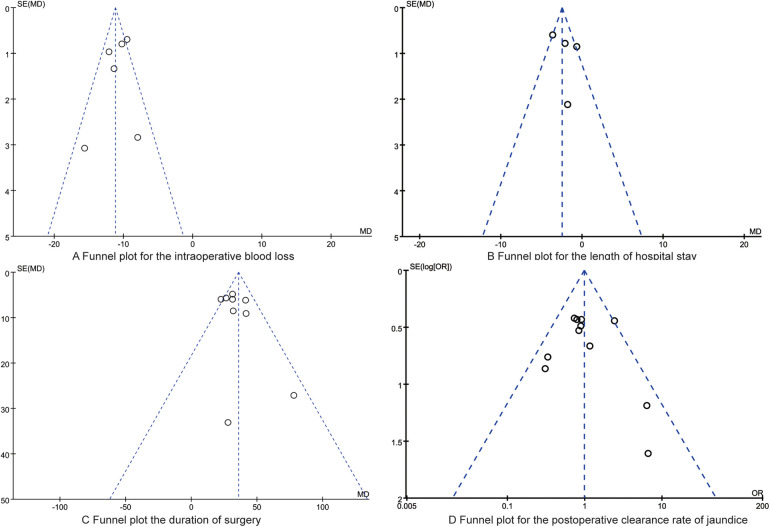
The funnel plots for the intraoperative blood loss **(A)**, the length of hospital stay **(B)**, the duration of surgery **(C)**, the postoperative clearance rate of jaundice **(D)**.

**Figure 6:**
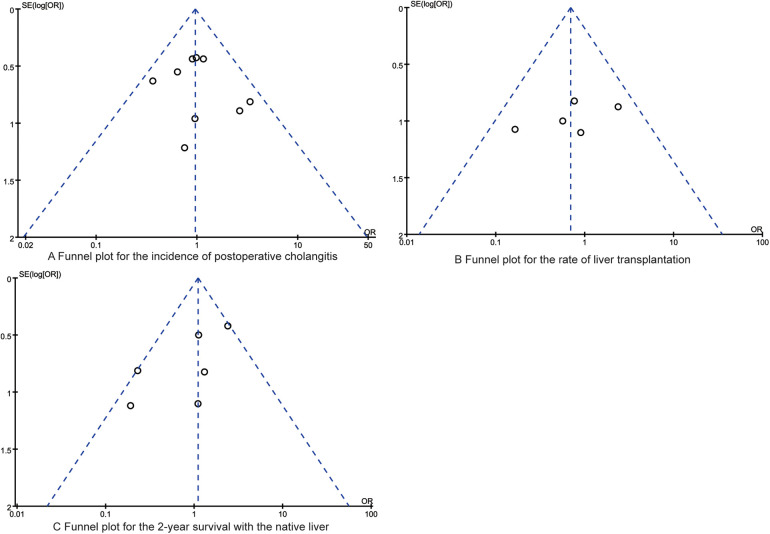
The funnel plots for the synthesized outcomes. **(A)** Funnel plot for the incidence of postoperative cholangitis; **(B)** Funnel plot for the rate of liver transplantation; **(C)** Funnel plot for the 2-year survival with the native liver.

Figures 5, 6 depict the distribution of data points within the funnel plots for each analysis, which are situated within the confines of an inverted funnel, exhibiting a uniform dispersion. This pattern infers a low probability of publication bias. The outcomes of the Egger's regression analysis further corroborate the absence of significant publication bias across all consolidated findings, with all *P*-values exceeding the threshold of 0.05.

## Discussion

Biliary atresia represents a form of biliary obstruction distinguished by its progressive inflammatory and fibrotic nature within the liver. The absence of timely intervention may culminate in the development of cholestatic cirrhosis, potentially progressing to liver failure and ultimately, mortality (34). Currently, the predominant surgical intervention for biliary atresia is the Kasai portoenterostomy, which aims to alleviate biliary obstruction through the anastomosis of the bile ducts to the jejunum. Despite the potential for significant improvement in biliary drainage, a subset of pediatric patients may still experience disease progression. This progression is attributed to the destruction of bile ducts and the development of liver fibrosis secondary to autoimmune processes, which, if unchecked, can culminate in the onset of liver cirrhosis (35). In the current era, with ongoing advancements in diagnostic methodologies, surgical procedures, and postoperative care, the 2-year survival rate for patients diagnosed with biliary atresia has been reported to be approximately 60% (36). The 5-year native liver survival rate among pediatric patients post-Kasai portoenterostomy ranges from 30% to 50%. Furthermore, it is noted that a subset, estimated at 15%–20%, maintains viable native liver function into adulthood (37). The body of evidence suggests that the expertise in the management of biliary atresia is a pivotal determinant of patient outcomes. Specifically, the rate of postoperative jaundice resolution serves as a critical metric for assessing the efficacy of Kasai portoenterostomy (38). Enhancing surgical proficiency and ameliorating pediatric outcomes represent significant avenues for research in the field of biliary atresia. The findings of this meta-analysis indicate that, when juxtaposed with the OPE approach, the LPE procedure achieves comparable rates of jaundice resolution and 2-year native liver survival. Additionally, LPE is associated with benefits such as reduced intraoperative blood loss and a diminished duration of hospital confinement, albeit with a longer operative time. These outcomes corroborate the results of prior studies (39, 40), underscoring the need for further investigation into the comparative effectiveness of these surgical modalities.

The increased blood loss in patients undergoing OPE can be attributed to a multitude of factors. Firstly, the surgical technique itself plays a role, as open surgeries typically require larger incisions that increase tissue exposure, thereby potentially leading to more blood loss. Additionally, the manual dissection process in OPE can result in greater tissue trauma and bleeding, particularly when compared to the more precise dissection that can be achieved with laparoscopic instruments. Furthermore, the magnified visualization afforded by laparoscopic surgery can enhance the identification and control of blood vessels, thereby reducing blood loss, a precision that is less readily achievable in open procedures (41). Lastly, postoperative complications such as hematomas or seromas, which are potentially more common following open surgeries, can also contribute to the increased blood loss observed in OPE patients (42). It is crucial to highlight that the quantification of blood loss in the surgical procedures of the included studies was primarily based on visual estimation by the surgeons. This method, while commonly employed, has been widely recognized for its inherent inaccuracy. Visual estimation by surgeons and anesthesiologists is known to be an unreliable metric, often leading to an overestimation of the actual blood loss. This overestimation can be attributed to several factors, including the difficulty in distinguishing blood from other fluids in the surgical field, such as irrigation fluids, lymph, and serum, which do not significantly alter the appearance of blood to the naked eye. Additionally, the pressure and pace of the operating environment may influence the surgeon's ability to accurately gauge the volume of blood lost. Consequently, the reported blood loss figures in our study may not reflect the true extent of blood loss, and this limitation should be taken into account when interpreting the results.

Within the hilar region, three fundamental types of microbiliary structures have been identified: bile ducts, bile ductal collecting ducts, and bile ductal glands. Notably, only those bile ducts that are in continuity with the intrahepatic biliary system are capable of facilitating bile drainage (43). The Kasai procedure involves the resection of the atretic fibrotic extrahepatic bile duct and the subsequent hepatoenterostomy, aiming to restore bile flow. Consequently, a thorough understanding of the liver hilum anatomy and the precise excision of the fibrous mass are paramount to the operative success. In the context of open Kasai surgery, the deep location of the hepatic hilum presents challenges in adequately exposing the fibrous tissue for resection (44). Utilizing a laparoscopic lens, direct visualization of the liver hilum is facilitated, enabling the magnification of the intricate hilar tissue structures through laparoscopy. Under laparoscopic guidance, the operative field is rendered clearly visible, allowing for distinct observation of the fibrous mass at the hilum and its adjacent tissues. This enhanced visualization aids the surgeon in the precise and meticulous hemostasis of minor hepatic arterial and portal venous branches, thereby improving the accuracy and finesse of the procedure (45). Furthermore, the minimally invasive nature of LPE, characterized by smaller incisions and reduced bleeding at the wound margins, constitutes one of its significant advantages (46). One of the primary postoperative complications associated with wound healing in classic laparotomies is the development of incisional hernia. Unfortunately, the studies included in our analysis did not provide specific data on the incidence of incisional hernia among patients who underwent OPE. This represents a significant limitation of our study, as understanding the occurrence of such complications could yield valuable insights into the comparative effectiveness of different surgical approaches. Therefore, future research is essential to provide a more comprehensive understanding of this complication within the context of OPE.

Previous studies (47, 48) have pointed out that minimizing thermal injury to the hilar microbile ducts during surgery is a critical factor in ensuring effective bile drainage, and it is imperative that this principle is consistently adhered to throughout the LPE procedure. Some surgeons mitigate the use of electrocautery by employing meticulous blunt and sharp dissection techniques. Additionally, the utilization of vascular ligation systems in the resection of portal vein branches and the dissection of the hepatic hilar fibrous mass edge can further diminish the potential thermal damage caused by electrocoagulation to the bile ducts (49). Some scholars (50) have employed a suspension technique to achieve comprehensive exposure of the liver hilum, subsequently magnifying the visual field with laparoscopic equipment. Upon removal of the fibrous plate, it has been observed that the open bile ducts or bile thrombi are more distinctly visualized. It is posited that preserving the hilar fibrous plate and utilizing superficial suture techniques under laparoscopic guidance may reduce iatrogenic injury to the exposed capillary bile ducts. In addition, some scholars (51) retained connective tissue can be strategically utilized for suturing purposes. This approach has dual benefits: it circumvents the challenge of performing unorganized sutures on the liver surface, and it eliminates the need for arterial and venous dissection, thereby minimizing the anastomotic area. Consequently, this technique simplifies the operative procedure. Additionally, the preservation of the fibrous plate is believed to prevent subsequent microbile duct occlusion that might arise from progressive inflammation, thus potentially enhancing bile excretion efficacy.

Laparoscopic Kasai surgery necessitates a profound foundation in open Kasai surgical procedures coupled with proficiency in laparoscopic techniques. It has been observed that after accruing experience through approximately 10 cases, the operative duration can be significantly reduced by approximately 50% (52). Significant heterogeneity is observed in key surgical outcomes, notably blood loss and operative duration in this meta-analysis. This variability is a complex phenomenon that may be attributed to a multitude of factors, with surgeon experience and patient-specific characteristics being two of the most influential. The skill and expertise of the surgeon can significantly impact both the amount of blood loss and the time taken to complete the surgery, as more experienced surgeons may employ refined techniques that minimize blood loss and optimize procedural efficiency (53). Patient differences, including but not limited to age, comorbidities, and preoperative health status, also play a crucial role in determining the surgical outcomes. These factors can influence the complexity of the procedure, the patient's response to anesthesia, and the overall tolerance to surgery, thereby affecting both blood loss and surgery time (54). A comprehensive understanding of these factors is essential for the accurate interpretation of the observed heterogeneity and for the development of strategies aimed at standardizing surgical practices to improve patient outcomes.

The surgical management of biliary atresia is characterized by its unique challenges, with the therapeutic outcomes not being immediately apparent in the postoperative short term. It is recognized that the efficacy of treatment for biliary atresia is multifactorial and not attributable to a single operative factor (55). The therapeutic efficacy in pediatric biliary atresia cases is intricately linked to the individual variability of the disease. It is contingent upon meticulous attention to detail during the operative procedures of dissection and anastomosis, as well as the quality of postoperative care and the management of cholangitis, both in terms of prevention and treatment (56). Surgeons specializing in biliary atresia must focus not only on refining the intricacies of the surgical procedure but also on the comprehensive management throughout the perioperative period. LPE offers the benefits of reduced tissue trauma and aesthetic incisions. By advancing technical approaches and leveraging specialized instruments, laparoscopic resection and reconstructive surgery can attain equivalent operative quality to that of OPE (57).

Although minimally invasive surgical techniques are typically lauded for their ability to shorten hospital stays due to reduced postoperative recovery times and complications, this advantage is not uniformly observed in the context of biliary atresia. Patients diagnosed with biliary atresia often find themselves necessitating an extended hospitalization period, which is primarily contingent upon the attainment of normalized liver function test results. This requirement for prolonged care is not a reflection of the surgical approach adopted but is instead intricately linked to the unique postoperative journey of each individual patient. The recovery course following biliary atresia surgery is highly variable and can be influenced by a complex interplay of factors (9). These factors may encompass the patient's overall health status, the presence of underlying comorbidities, the degree of liver damage prior to surgery, and the body's response to the surgical intervention. Additionally, postoperative care, including medical management, nutritional support, and the prevention of complications such as infection, plays a critical role in determining the duration of hospital stay (7, 58). It is also worth considering that the complexity of biliary atresia as a disease entity may demand closer monitoring and a more cautious discharge planning process. This is to ensure that the patient's condition remains stable and that they have the necessary support and resources in place for a smooth transition back to their home environment. Therefore, while the surgical approach may contribute to the overall treatment plan, it is the patient's specific circumstances and response to treatment that ultimately dictate the length of their hospitalization.

This meta-analysis is subject to several limitations that merit consideration. Firstly, we acknowledge a significant limitation pertaining to the evidence base, which is predominantly constituted by observational studies. The absence of a substantial number of high-quality randomized controlled trials (RCTs) introduces potential biases into our analysis, as these biases can arise from various sources, including but not limited to, surgeon experience and patient-specific differences. Secondly, in our meta-analysis, we primarily assessed short-term outcomes like jaundice clearance and 2-year survival rates, neglecting the long-term effects on liver function and quality of life due to limited collected data. Future research should focus on longitudinal studies to elucidate the enduring impact of surgical interventions and prioritize RCTs for a more definitive evidence. Thirdly, we observed significant heterogeneity across several of the outcomes analyzed. However, due to limitations in the available data, we were unable to conduct subgroup analyses, which would have been necessary to explore this heterogeneity more deeply. Furthermore, while the impact of Roux limb length on surgical outcomes is an important factor that merits investigation, our capacity to analyze this variable was hindered by the lack of relevant data in the studies included in our analysis. There is a clear necessity for future research endeavors to meticulously investigate the role of Roux limb length in shaping postoperative outcomes for patients who have undergone the Kasai procedure. Finally, the majority of the studies encompassed within our analysis did not provide specific information on the incidence of native liver survivors or the number of patients who were scheduled for programmed liver transplantation. This is indeed a significant aspect that warrants detailed examination, as it pertains to the long-term outcomes and clinical management of children with biliary atresia. Future research should address this gap in order to provide a more holistic understanding of the treatment outcomes and the decision-making processes surrounding liver transplantation in children with biliary atresia.

## Conclusion

In summary, the pooled data from this meta-analysis indicate that LPE is equivalent to OPE in achieving resolution of jaundice and maintaining a 2-year native liver survival rate. Furthermore, LPE offers additional benefits, such as reduced intraoperative blood loss and a shorter hospital stay, although it is associated with a longer operative duration. These results imply that LPE may provide better outcomes in the management of biliary atresia and may be preferentially considered in clinical surgical decision-making processes. However, caution is advised in interpreting these findings due to the limited number of studies included in this analysis. Future validation through additional high-quality studies is necessary to confirm these preliminary results.

## Data Availability

The original contributions presented in the study are included in the article/Supplementary Material, further inquiries can be directed to the corresponding author.
